# The effect of concentration, reconstitution solution and pH on the stability of a remifentanil hydrochloride and propofol admixture for simultaneous co-infusion

**DOI:** 10.1186/s12871-020-01194-5

**Published:** 2020-11-12

**Authors:** Emily Henkel, Rebecca Vella, Kieran Behan, David Austin, Peter Kruger, Andrew Fenning

**Affiliations:** 1 Central Queensland University, School of Health, Medical and Applied Sciences, 554-700 Yaamba Road, Rockhampton, QLD 4701 Australia; 2 Department of Pharmacy and Intensive Care Unit, Rockhampton Hospital, Canning Street, Rockhampton, QLD 4700 Australia; 3 Intensive Care Unit, Rockhampton Hospital, Canning Street, Rockhampton, QLD 4700 Australia; 4 Intensive Care Unit, Princess Alexandra Hospital, 199 Ipswich Road, Woolloongabba, QLD 4102 Australia

**Keywords:** Chemical stability, Drug-drug interaction(s), HPLC, Pharmaceutical preparations, Propofol, Remifentanil

## Abstract

**Background:**

There are scenarios where pre-mixing and infusing analgesic and anaesthetic agents as a single intravenous (IV) solution is highly desirable; however, it is important to ensure the agents are compatible when mixed. As such, the long-term stability of a remifentanil-propofol mixture, and means of improving this, were assessed across a range of remifentanil concentrations, diluents, and time points.

**Methods:**

Remifentanil was reconstituted with ultrapure water, 0.9% saline, 20% saline, or 8.4% sodium bicarbonate solution (the latter two chosen for their pH characteristics, rather than their use in pharmaceutical reconstitution) and then mixed with propofol (1%) or further diluted with water to derive concentrations of 10–50 μg mL^− 1^. Remifentanil and propofol concentrations were determined initially and then periodically for up to 24 h using high performance liquid chromatography (HPLC). Mass spectrometry (MS) was used to detect degradation products in solutions containing 30 μg mL^− 1^ of remifentanil. Statistical analysis was performed using ANOVA and Student’s *t*-test, with a significance value of 0.05.

**Results:**

Isolated remifentanil (pH < 4) and propofol (pH 7.35) did not degrade significantly when reconstituted with water or saline solution over 24 h, while remifentanil reconstituted with sodium bicarbonate degraded significantly (*P* < 0.001, pH 8.65). Mixing with propofol substantially increased the pH of the mixture and resulted in significant remifentanil degradation for all reconstitution solutions used, while propofol remained stable (pH 6.50). The amount of degradation product detected in samples containing isolated remifentanil and a mixture of the drugs was proportional to the remifentanil degradation observed.

**Conclusions:**

Remifentanil stability is affected by both the reconstitution solution used and when mixed with propofol, with pH appearing to be a contributing factor to degradation. If the pH of the solution and concentration of remifentanil are correctly controlled, e.g. through the use of a more acidic diluent, an admixture of remifentanil and propofol may be useful clinically.

**Supplementary Information:**

**Supplementary information** accompanies this paper at 10.1186/s12871-020-01194-5.

## Background

Optimisation of both analgesia and sedation is vital to ensure adequate pain control, minimise agitation and anxiety, facilitate patient compliance for mechanical ventilation or diagnostic interventions, and provide patient comfort [1]. Most patients receiving invasive ventilation are on several continuous infusions, often achieved using separate or multi-channel infusion pumps, IV connectors, and/or multi-lumen catheters [2]. Even in well-equipped hospital settings (e.g. intensive care units) this can be a complex undertaking, with numerous potential correlated patient safety risks such as separate drugs running at incorrect infusion rates and required medications being connected to the infusion system but not administered to the patient [3]. However, there are scenarios outside of a sophisticated medical facility environment where the availability of specialised equipment may be limited, or many patients must be attended to within a short schedule. This can include those in rural or remote locations, a busy ambulatory surgery centre or office practice, or military medical personnel attending to wounded soldiers in harsh environments [4, 5]. In all instances, it may be advantageous to pre-mix infusion agents and administer them via a simplified, single IV infusion. The kinetics of such infusions is not always well-understood.

Remifentanil is a highly potent analgesic agent. It is an ultra-short-acting mu-opioid receptor agonist that undergoes organ independent metabolism by blood and non-specific tissue esterases, forming an inactive metabolite [6, 7]. It has a rapid onset of effect, with maximum ventilatory depression occurring approximately 2–3 min following administration of an initial bolus [8]. The context sensitive half-life (the time taken for blood concentration to decrease by 50% following termination of a continuous infusion that maintained a steady-state concentration) is around 3 min even after prolonged infusions, with no significant drug accumulation [9, 10]. This is a point of difference from other commonly used analgesic agents, where the duration of infusion or renal impairment may impact on duration of effect. This pharmacokinetic profile may be clinically advantageous in a variety of patients.

Propofol is a commonly-used short-acting sedative agent with rapid onset of anaesthesia (only a few seconds), duration of effect (3–5 min), and recovery [11]. The mechanism of action is via positive modulation of the inhibitory function of the gamma-aminobutyric acid (GABA) neurotransmitter through GABA_A_ receptors [12]. Using a combination of remifentanil and propofol can offer several advantages in a clinical setting [13, 14]. The short duration of pharmacological action shared by remifentanil and propofol may allow for improved control over pain and anaesthesia management and afford faster recovery for patients, while the synergistic relationship exhibited when the drugs are co-administered reduces remifentanil and propofol requirements [4, 14].

Manufacturers of remifentanil advise against mixing with propofol; however, further explanation is not provided. The utility of a remifentanil-propofol admixture has already been explored in areas such as radiation therapy, dental extraction, and paediatric and elective outpatient surgeries, while a current clinical trial is investigating the use of a remifentanil-propofol mixture for breast cancer surgery [4, 15–19]. Previous studies have concluded that while simultaneous infusion removed the ability to selectively control the use of each drug, it resulted in decreased incidences of procedural respiratory depression and patient recovery time [18, 19]. Furthermore, when mixed with propofol, remifentanil has been found to inhibit the bacterial growth that readily occurs within the lipid emulsion, possibly due to its glycine excipient and low pH [20, 21].

When admixing remifentanil and propofol for simultaneous infusion, it is crucial to understand their compatibility from a chemical perspective, including the effect of any interactions on stability and efficacy. If degradation unknowingly occurs, an unpredictable response may arise and patient care may be compromised. Research in this area could potentially lead to methods of improving the stability of the mixture and ensure its safety and effectiveness, in addition to providing avenues for the use of other combinations of opiate agonists and short-acting anaesthetics. As propofol is an opaque liquid, it is difficult to detect incompatibility or any changes in solution stability through visual assessment alone. In addition, the organ-independent metabolism of remifentanil combined with its short duration of effect mandate careful evaluation of stability of the parent compound in any mixture or co-infusion.

Previous studies have, to different degrees, considered factors such as the storage vessel and drug concentration when exploring the stability of a remifentanil and propofol mixture; most investigations include admixtures containing two or three concentrations of remifentanil and/or propofol that are stored in polyvinyl chloride and propylene vessels [22, 23]. For mixtures of remifentanil and propofol specifically, there are a lack of studies investigating a variety of remifentanil concentrations, the impact of mixing on both remifentanil and propofol concentration, and how manipulating solution pH (through the use of different remifentanil reconstitution mediums) affects the stability of the mixture. pH is of particular importance for a combination of remifentanil and propofol as remifentanil is believed to undergo rapid hydrolysis when exposed to a pH range of 7–7.5 [21]. After reconstitution with water, remifentanil has a pH of 3.0 [24]. In comparison, propofol can have a pH ranging from 6 to 8.5 [25].

The aim of this study was to determine the stability of both remifentanil and propofol solutions, alone and in combination, when stored in glass over 24 h, and ascertain if drug concentration, diluent used, or pH could be altered to improve their stability from a pharmaceutic perspective. This could indicate if remifentanil and propofol are compatible to be pre-mixed and infused as a single intravenous solution.

## Methods

### Materials and reagents

Remifentanil hydrochloride (“Ultiva for Injection” from GlaxoSmithKline Australia Pty Ltd., Boronia, VIC, Australia, and “DBL Remifentanil powder for injection” from Hospira Pty Ltd., Melbourne, VIC, Australia), Propofol (containing propofol (10 mg mL^− 1^), soya oil (100 mg mL^− 1^), glycerol (22.5 mg mL^− 1^), egg lecithin (12 mg mL^− 1^), sodium oleate (0.3 mg mL^− 1^); “Propofol Sandoz” from Sandoz Pty Ltd., Pyrmont, NSW, Australia, and “Provive 1%” from Claris Lifesciences (Aust) Pty Limited, Burwood, NSW, Australia), 0.9% saline solution, 20% saline solution and 8.4% sodium bicarbonate solution were all of clinical grade and donated by the Rockhampton Hospital Pharmacy Department (Rockhampton, QLD, Australia). Methanol, acetonitrile and ammonium acetate were purchased from Thermo Fisher Scientific (Scoresby, VIC, Australia). All chemicals were ACS analytical grade or HPLC grade. Ultrapure water was prepared by a Milli-Q® Reference Water Purification System (Merck Millipore, Bayswater, VIC, Australia).

### Instrumentation

All samples were analysed using an Agilent Technologies 1200 series HPLC (Agilent Technologies, Melbourne, VIC, Australia) equipped with a variable wavelength diode array detector set at 210 nm and 270 nm for remifentanil and propofol analysis, respectively. The remifentanil protocol used an Agilent Eclipse XDB-C18 column with dimensions of 150 × 4.6 mm with a particle size of 5 μm, and a mobile phase of 75% methanol and 25% 10 mM ammonium acetate (flow rate 1.5 mL min^− 1^) [26–28]. The protocol for propofol analysis used an Agilent Eclipse XDB-C18 column with dimensions of 250 × 4.6 mm and a particle size of 5 μm. The mobile phase consisted of 65% acetonitrile and 35% water with a flow rate of 2.0 mL min^− 1^ [29, 30]. Solution pH was determined using a Eutech Instruments 700 pH meter (Eutech Instruments Pte Ltd., Singapore). Subsequent assays were performed on the same samples at 1, 2, 3, 4, 6, 12 and 24 h following preparation.

For HPLC-MS analysis, a Prominence HPLC system (Shimadzu Scientific Instruments, Rydalmere, NSW, Australia) coupled to an API3200 LC-MS/MS mass spectrometer (Applied Biosystems/MDS Analytical Technologies/SCIEX, Mt Waverley, VIC, Australia) was utilised. Separation was achieved using an Agilent Zorbax SB C18 column with dimensions of 150 × 4.6 mm and a particle size of 5 μm, and a mobile phase of 60% methanol and 40% 10 mM ammonium acetate (flow rate 1.3 mL min^− 1^). The MS system was run in the positive ion mode using nitrogen as the desolvation gas.

### Sample preparation

Triplicate 5 mg preparations of remifentanil hydrochloride for injection were reconstituted with 5 mL of either ultrapure water, 0.9% saline solution, 20% saline solution, or 8.4% sodium bicarbonate solution (the latter two chosen for their pH characteristics, rather than their use in pharmaceutical reconstitution). Samples were then added to 10 mg mL^− 1^ propofol for injection (final propofol concentration of 9.5 mg mL^− 1^) or left in isolation by mixing with ultrapure water to produce a solution with a final remifentanil concentration of 50 μg mL^− 1^. This procedure was repeated to give solutions with final remifentanil concentrations of 40, 30, 20 and 10 μg mL^− 1^ (final propofol concentrations of 9.6, 9.7, 9.8 and 9.9 mg mL^− 1^).

To determine propofol degradation in isolation, triplicate volumes of 1–5 mL of ultrapure water, 0.9% saline solution and 20% saline solution were added to 10 mg mL^− 1^ propofol for final propofol concentrations of 9.5–9.9 mg mL^− 1^.

Immediately following preparation and pH determination, the remifentanil and propofol concentration in each sample was assessed in emulsion using HPLC. Samples demonstrating significant remifentanil deterioration were analysed further for the presence of degradation products using HPLC-MS. Subsequent assays were taken 1, 2, 3, 4, 6, 12 and 24 h following preparation. All samples were stored at room temperature (22 °C – 24 °C) between assays, and inverted prior to aliquot removal to prevent mixture separation and reduce the influence of oil droplet flocculation/creaming [31, 32].

### Statistical analysis

Statistical analysis was performed using ANOVA and Student’s *t*-test where appropriate, with results deemed significant when *P* ≤ 0.05 (Prism version 4.02; GraphPad Software, San Diego, CA, USA).

## Results

### Remifentanil in isolation

**Fig. 1:**
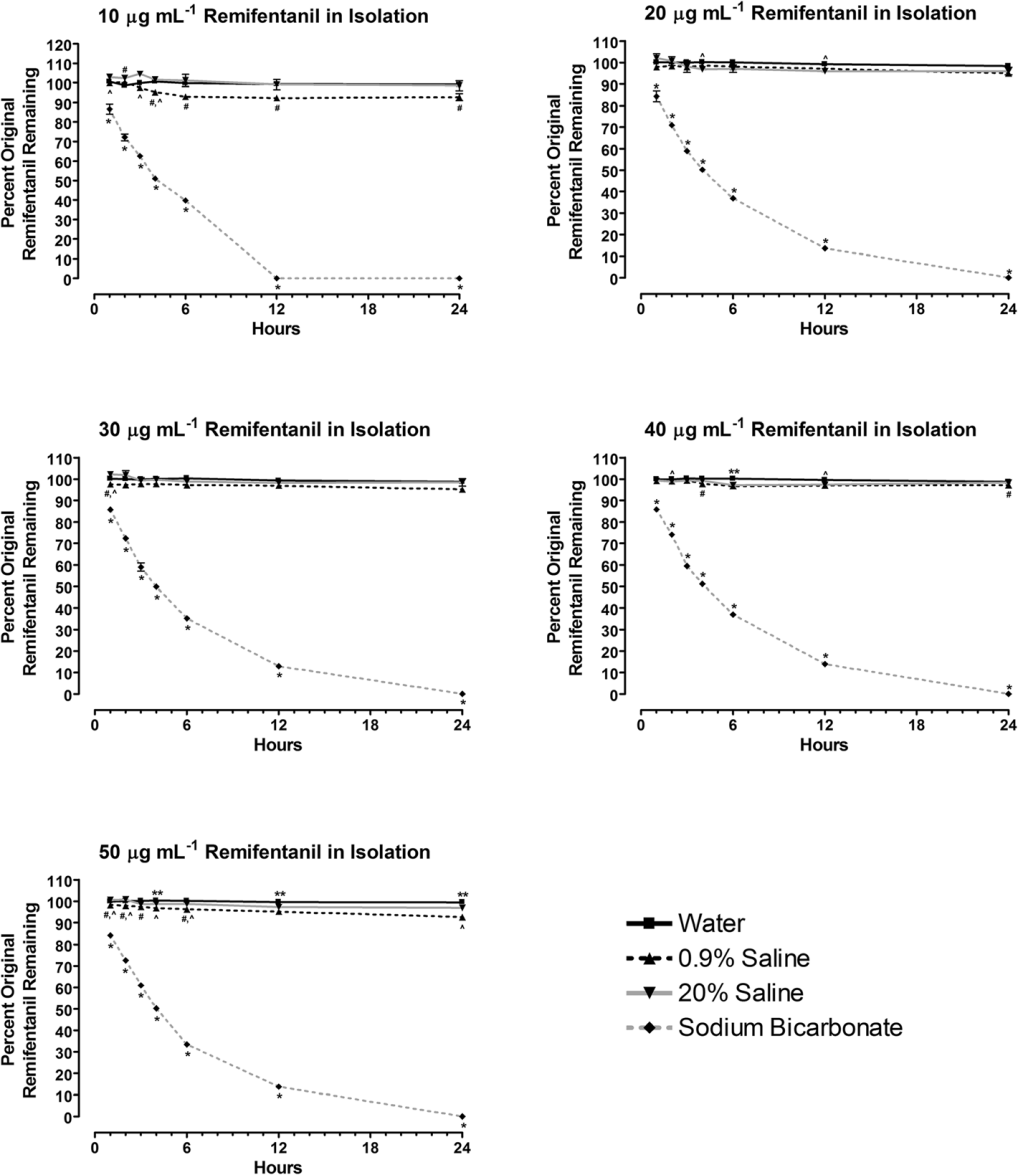
Percent original remaining for 10 μg mL^− 1^, 20 μg mL^− 1^, 30 μg mL^− 1^, 40 μg mL^− 1^ and 50 μg mL^− 1^ remifentanil reconstituted with water, 0.9% saline, 20% saline and sodium bicarbonate solution and left in isolation over 24 h. Data expressed as mean ± SEM, *n* = 3. *P < 0.01 vs. water, 0.9% saline and 20% saline; ^#^*P* < 0.04 vs. water; ^^^*P* < 0.05 vs. 20% saline; **P < 0.05 vs. 0.9% saline and 20% saline

No obvious precipitate was formed in any of the remifentanil solutions over time. Remifentanil in isolation did not degrade significantly over 24 h when reconstituted with either water, 0.9% saline solution or 20% saline solution; all concentrations contained more than 92% of the original remifentanil after 24 h (Fig. 1). The pH of these solutions over 24 h were also similar, averaging 3.74, 3.94, and 3.95 for remifentanil reconstituted with water, 0.9% saline and 20% saline, respectively (see Additional file 1).

Remifentanil reconstituted with sodium bicarbonate solution degraded rapidly, with no remifentanil remaining after 24 h (Fig. 1). Furthermore, compared to the other reconstitution solutions, sodium bicarbonate had significant effects on remifentanil degradation (*P* < 0.01) after only 1 h for all concentrations. The pH of the sodium bicarbonate solutions averaged 8.65 over time (see Additional file 1).

### Propofol in isolation

Propofol did not degrade significantly over 24 h when in isolation or after the addition of water, 0.9% saline solution, or 20% saline solution, with all solutions having more than 97% of the original propofol remaining after 24 h (Table 1). There were no obvious visual signs of propofol emulsion instability or separation following the addition of the diluents over the time period tested. The pH of propofol in isolation and after mixing with water did not change over 24 h (pH = 7.70). These solutions had a significantly greater pH than propofol mixed with 0.9% saline solution (average pH of 7.40, *P* < 0.0001) and 20% saline solution (average pH of 6.98, P < 0.0001) (Table 1).

**Table 1:** pH and percentage of the original propofol remaining when mixed with water and saline solution

	10 μg mL^**−** **1**^				20 μg mL^**−** **1**^				30 μg mL^**−** **1**^				40 μg mL^**−** **1**^				50 μg mL^**−** **1**^			
	1 h	6 h	12 h	24 h	1 h	6 h	12 h	24 h	1 h	6 h	12 h	24 h	1 h	6 h	12 h	24 h	1 h	6 h	12 h	24 h
**Percent Propofol Remaining when left in Isolation (mean ± SEM;** ***n*** **= 3)**																				
**I**	102.3 ± 0.90	101.1 ± 1.03	100.5 ± 1.05	100.2 ± 1.04	102.3 ± 0.90	101.1 ± 1.03	100.5 ± 1.05	100.2 ± 1.04	102.3 ± 0.90	101.1 ± 1.03	100.5 ± 1.05	100.2 ± 1.04	102.3 ± 0.90	101.1 ± 1.03	100.5 ± 1.05	100.2 ± 1.04	102.3 ± 0.90	101.1 ± 1.03	100.5 ± 1.05	100.2 ± 1.04
**W**	101.1 ± 0.22	100.4 ± 2.37	100.6 ± 2.00	99.6 ± 1.94 *	102.2 ± 2.27	102.1 ± 1.92	101.8 ± 1.48	101.6 ± 1.58	99.8 ± 0.20 *a*	100.5 ± 0.54 *d*	100.9 ± 0.35 *d*	99.5 ± 0.72	101.6 ± 2.06	100.5 ± 1.80	100.3 ± 1.69	99.4 ± 1.56	99.8 ± 0.13 * a	98.7 ± 0.21 *	98.2 ± 0.13	98.0 ± 0.21
**0.9%**	99.9 ± 0.48	99.4 ± 0.48 *b*	98.6 ± 0.18 * b	98.3 ± 0.27	100.5 ± 0.07 * d	101.9 ± 0.15 *	100.5 ± 0.39	99.1 ± 0.33	101.2 ± 0.32 ** *d*	100.7 ± 0.89	99.5 ± 0.54	97.9 ± 0.12 *b*	101.1 ± 0.78	98.9 ± 0.86 *b*	97.9 ± 0.60 * b	98.0 ± 0.54 *	99.4 ± 0.12 #	98.8 ± 0.28 * b	98.1 ± 0.29 *b*	98.0 ± 0.06 *b*
**20%**	100.0 ± 0.49	100.0 ± 0.48 *d*	99.3 ± 0.06 *d*	98.1 ± 0.10 *b*	100.1 ± 0.12	99.7 ± 0.48 *d*	99.3 ± 0.24 *d*	98.9 ± 0.15	98.7 ± 0.58 #	100.8 ± 0.59 *d*	100.1 ± 0.83	99.0 ± 0.88	99.5 ± 0.19 #	100.7 ± 0.16 *d*	101.3 ± 0.37 *a, b, d*	100.9 ± 0.32 *a, b, d*	99.1 ± 0.15 # *b*	97.9 ± 0.13 #	97.9 ± 0.33	97.8 ± 0.41
**pH of Solution (mean ± SEM; n = 3)**																				
**I**	7.69 ± 0.01	7.70 ± 0.02	7.76 ± 0.01	7.74 ± 0.00	7.69 ± 0.01	7.70 ± 0.02	7.76 ± 0.01	7.74 ± 0.00	7.69 ± 0.01	7.70 ± 0.02	7.76 ± 0.01	7.74 ± 0.00	7.69 ± 0.01	7.70 ± 0.02	7.76 ± 0.01	7.74 ± 0.00	7.69 ± 0.01	7.70 ± 0.02	7.76 ± 0.01	7.74 ± 0.00
**W**	7.69 ± 0.01	7.72 ± 0.03 *d*	7.80 ± 0.03	7.78 ± 0.03	7.74 ± 0.01 #	7.78 ± 0.02 *d*	7.83 ± 0.03	7.81 ± 0.02	7.76 ± 0.01 #	7.79 ± 0.02 *d*	7.85 ± 0.02	7.82 ± 0.03 #	7.70 ± 0.01	7.75 ± 0.02 *d*	7.80 ± 0.03	7.78 ± 0.03	7.67 ± 0.02	7.61 ± 0.02 #	7.65 ± 0.05	7.67 ± 0.03
**0.9%**	7.65 ± 0.01	7.52 ± 0.03 ^	7.48 ± 0.00 ^	7.43 ± 0.01 ^	7.47 ± 0.04 ^ *a, d*	7.46 ± 0.02 ^	7.46 ± 0.02 ^	7.36 ± 0.01 ^ *a*	7.44 ± 0.02 ^ *a, d*	7.45 ± 0.02 ^	7.52 ± 0.04 ^	7.34 ± 0.01 ^ *a*	7.42 ± 0.02 ^ *a, d*	7.21 ± 0.02 ^ *a,b,c*	7.26 ± 0.02 ^ *a,b,c*	7.24 ± 0.02 ^ *a,b,c*	7.22 ± 0.01 ^ *a*	7.23 ± 0.02 ^ *a,b,c*	7.21 ± 0.01 ^ *a,b,c*	7.20 ± 0.02 ^ *a,b,c*
**20%**	7.09 ± 0.02 ^	7.02 ± 0.00 ^	7.04 ± 0.01 ^	7.07 ± 0.02 ^	7.02 ± 0.02 ^	6.95 ± 0.01 ^ *a*	6.98 ± 0.00 ^ *a*	7.01 ± 0.01 ^	6.94 ± 0.01 ^ *a, b*	6.98 ± 0.02 ^	6.98 ± 0.01 ^ *a*	6.98 ± 0.01 ^ *a*	6.93 ± 0.02 ^ *a, b*	6.96 ± 0.01 ^ *a*	6.95 ± 0.01 ^ *a, b*	6.91 ± 0.01 ^ *e*	6.92 ± 0.02 ^ *a, b*	6.94 ± 0.00 ^ *a*	6.97 ± 0.00 ^ *a, b*	6.94 ± 0.00 ^ *a,b,c*

I = in isolation, W = water, 0.9% = 0.9% saline, 20% = 20% saline

*P < 0.05 vs. 20% saline solution; ^**^P < 0.03 vs. water and 20% saline solution; ^#^*P* < 0.04 vs. propofol in isolation; ^^^*P* < 0.05 vs. all other reconstitution solutions

^a^P < 0.03 vs. 10 μg mL^− 1^; ^b^P < 0.04 vs. 20 μg mL^− 1^; ^c^P < 0.02 vs. 30 μg mL^− 1^; ^d^P < 0.04 vs. 50 μg mL^− 1^; ^e^P < 0.05 vs. all other concentrations

### Remifentanil-propofol mixture

There were no obvious visual incompatibilities or signs of emulsion instability/separation when remifentanil and propofol were mixed over the time period tested.

Remifentanil showed significant degradation when mixed with propofol. The percentage of remifentanil remaining after reconstituting with water or 0.9% saline and then mixing with propofol decreased by 50–60% over 24 h, compared to the same remifentanil solutions in isolation. These solutions also had the highest pH readings over 24 h, with averages of 6.86 for 0.9% saline-reconstituted solutions and 6.96 for water-reconstituted solutions (Table 2). Propofol in these solutions, as well as those containing remifentanil reconstituted with 20% saline solution, remained stable, with all solutions having greater than 96% of the original propofol remaining after 24 h (Table 2).

**Table 2:** pH and percentage of the original remifentanil/propofol remaining over 24 h when mixed

	10 μg mL^**−** **1**^				20 μg mL^**−** **1**^				30 μg mL^**−** **1**^				40 μg mL^**−** **1**^				50 μg mL^**−** **1**^			
	1 h	6 h	12 h	24 h	1 h	6 h	12 h	24 h	1 h	6 h	12 h	24 h	1 h	6 h	12 h	24 h	1 h	6 h	12 h	24 h
**Percent Remifentanil Remaining in Remifentanil-Propofol Mixture (mean ± SEM; n = 3)**																				
**W**	99.5 ± 2.40	81.5 ± 1.58	64.7 ± 2.49	43.3 ± 2.02	100.3 ± 3.47	84.4 ± 4.41	67.0 ± 2.34	46.7 ± 2.82	97.9 ± 0.72	83.1 ± 2.94	67.4 ± 3.42	48.2 ± 5.09	97.3 ± 0.63	81.7 ± 1.43 ^	66.0 ± 2.56 ^	45.3 ± 2.89 ^	97.7 ± 0.18	83.1 ± 0.53 ^	67.0 ± 0.73	46.9 ± 1.22
**0.9%**	93.5 ± 0.61	70.5 ± 1.24 *	53.8 ± 1.15 *	35.0 ± 0.49 * d	96.7 ± 0.95	74.1 ± 1.11 *d*	58.6 ± 1.29 * a, d	35.9 ± 1.91 * d	96.0 ± 0.49	78.0 ± 0.85 *a, b*	61.2 ± 1.36 *a*	37.4 ± 1.34 *d*	94.4 ± 0.74 *	74.9 ± 0.98 * a, d	56.7 ± 1.19 * d	34.8 ± 1.56 * d	95.6 ± 0.83	79.7 ± 1.01 * a	65.6 ± 1.26 *a*	46.2 ± 1.08
**20%**	96.6 ± 2.02	80.8 ± 2.08	63.3 ± 1.13	45.2 ± 2.30	96.0 ± 0.43	79.6 ± 0.86	66.3 ± 0.51	48.9 ± 1.93	96.6 ± 1.10	83.9 ± 0.84 *b*	71.4 ± 0.24 # *a, b*	53.5 ± 0.58 # *a*	97.5 ± 0.36	86.0 ± 0.34 *b*	75.0 ± 1.01 *a, b, c*	56.7 ± 0.37 *a, b, c*	98.8 ± 0.22 *	86.8 ± 0.67 *b*	74.8 ± 0.80 * a, b, c	60.0 ± 1.41 * a, b, c
**Percent Propofol Remaining in Remifentanil-Propofol Mixture (mean ± SEM; n = 3)**																				
**W**	99.6 ± 0.21 *c*	99.2 ± 0.21 *d*	98.7 ± 0.20 *b*	97.9 ± 0.21 *c*	99.5 ± 0.16 *c*	100.0 ± 0.21 *d*	99.4 ± 0.10 ^ *d*	98.3 ± 0.38 *c*	100.6 ± 0.00 ^	99.7 ± 0.65	99.4 ± 0.32 *d*	99.5 ± 0.17 ^	99.6 ± 0.19 *c*	99.3 ± 0.40	99.0 ± 0.12 *d*	97.8 ± 0.02 *c*	100.1 ± 0.16 *c*	98.0 ± 0.28 #	96.9 ± 0.67 #	97.9 ± 0.17 *c*
**0.9%**	100.8 ± 0.62	99.2 ± 0.58	98.6 ± 0.09	96.8 ± 0.97	98.0 ± 0.86	99.9 ± 0.53	98.0 ± 0.65	97.2 ± 0.59	98.6 ± 0.84	100.5 ± 1.80	100.5 ± 1.46	99.1 ± 1.58	100.1 ± 0.26	99.7 ± 0.15	98.3 ± 0.33	97.7 ± 0.06	99.6 ± 0.24	99.6 ± 0.11	99.3 ± 0.49	98.7 ± 0.49
**20%**	99.8 ± 0.13	99.4 ± 0.62	98.6 ± 0.42	99.3 ± 0.49	99.0 ± 0.27	98.4 ± 0.74	97.7 ± 0.49	97.3 ± 0.79	99.5 ± 0.28	98.1 ± 0.89	97.9 ± 0.84	96.5 ± 0.64 *a*	99.5 ± 0.24	99.4 ± 0.22	98.4 ± 0.35	97.2 ± 0.25 *a*	99.2 ± 0.32	98.9 ± 0.31	98.5 ± 0.46	96.5 ± 0.86 *a*
**pH of Solution (mean ± SEM;** ***n*** **= 6)**																				
**W**	7.42 ± 0.12	7.41 ± 0.10	7.42 ± 0.12 ^	6.72 ± 0.40	7.19 ± 0.14 *d*	7.18 ± 0.11	7.12 ± 0.13	6.66 ± 0.33	7.02 ± 0.16	6.98 ± 0.13 *a*	6.95 ± 0.16 *a*	6.55 ± 0.36	6.88 ± 0.20 *a*	6.89 ± 0.18 *a*	6.87 ± 0.18 *a*	6.31 ± 0.43	6.59 ± 0.23 *a*	6.67 ± 0.20 *a*	6.67 ± 0.23 *a*	6.20 ± 0.41
**0.9%**	7.17 ± 0.04	7.17 ± 0.05	7.13 ± 0.09	6.63 ± 0.33	7.10 ± 0.08	7.05 ± 0.06	6.94 ± 0.06	6.64 ± 0.26	6.87 ± 0.08 *a*	6.89 ± 0.07 *a*	6.75 ± 0.06 *a*	6.48 ± 0.28	6.68 ± 0.10 *a, b*	6.74 ± 0.09 *a, b*	6.66 ± 0.10 *a, b*	6.12 ± 0.35	6.44 ± 0.16 *a, b, c*	6.49 ± 0.14 *a, b, c*	6.47 ± 0.15 *a, b*	6.13 ± 0.33
**20%**	7.01 ± 0.01 *	7.00 ± 0.01 *	6.94 ± 0.03	6.36 ± 0.31	6.79 ± 0.02 * a	6.79 ± 0.02 * a	6.69 ± 0.08 *, a	5.95 ± 0.39	6.64 ± 0.02 * a, b	6.66 ± 0.01 * a, b	6.62 ± 0.03 *a*	5.84 ± 0.38	6.51 ± 0.05 *e*	6.53 ± 0.04 *e*	6.48 ± 0.06 *a*	5.72 ± 0.41	6.30 ± 0.06 *a, b, c*	6.34 ± 0.06 *a, b, c*	6.33 ± 0.08 *a, b, c*	5.71 ± 0.36

W = water, 0.9% = 0.9% saline, 20% = 20% saline

^*^P < 0.05 vs. all other reconstitution solutions; ^#^P < 0.05 vs. 0.9% saline solution; ^^^P < 0.05 vs. 20% saline solution

^a^P < 0.05 vs. 10 μg mL^− 1^; ^b^P < 0.05 vs. 20 μg mL^− 1^; ^c^P < 0.05 vs. 30 μg mL^− 1^; ^d^P < 0.05 vs. 50 μg mL^− 1^; ^e^P < 0.05 vs. all other concentrations

Concentration did not impact on the stability of water-reconstituted remifentanil mixed with propofol. Solutions containing 30 μg mL^− 1^ of remifentanil had significantly more propofol than all others after 24 h (*P* < 0.05), but this difference was only 1.2% greater than the next highest concentration (Table 2). For remifentanil reconstituted with 0.9% saline and mixed with propofol, a concentration of 50 μg mL^− 1^ was significantly more stable (*P* < 0.01) than every other concentration after 24 h (Table 2), with significant differences (*P* < 0.03) apparent between 50 μg mL^− 1^ and 10, 20 and 40 μg mL^− 1^ concentrations after 6 h. Solutions containing 50 μg mL^− 1^ remifentanil also had the lowest average pH over 24 h of 6.50. Remifentanil concentration had no statistically significant effect on propofol degradation in 0.9% saline mixtures (Table 2).

Remifentanil reconstituted with 20% saline and mixed with propofol showed the least degradation compared to the same remifentanil solutions in isolation, with 46–60% of the original remaining after 24 h. These solutions also had significantly lower (*P* < 0.0001) pH readings of all reconstitution solutions tested, with an overall average of 6.60 over 24 h (Table 2). Similarly, the 20% saline mixtures with the most stable remifentanil concentrations also had the lowest average pH over 24 h; solutions containing 40 and 50 μg mL^− 1^ of remifentanil were significantly more stable (P < 0.03) than those with 10, 20 and 30 μg mL^− 1^ from 12 h onwards (Table 2). However, propofol in mixtures with 30, 40 and 50 μg mL^− 1^ of remifentanil reconstituted with 20% saline solution were significantly less stable than those containing 10 μg mL^− 1^ of remifentanil after 24 h (*P* < 0.05) (Table 2).

### Remifentanil degradation product

**Fig. 2:**
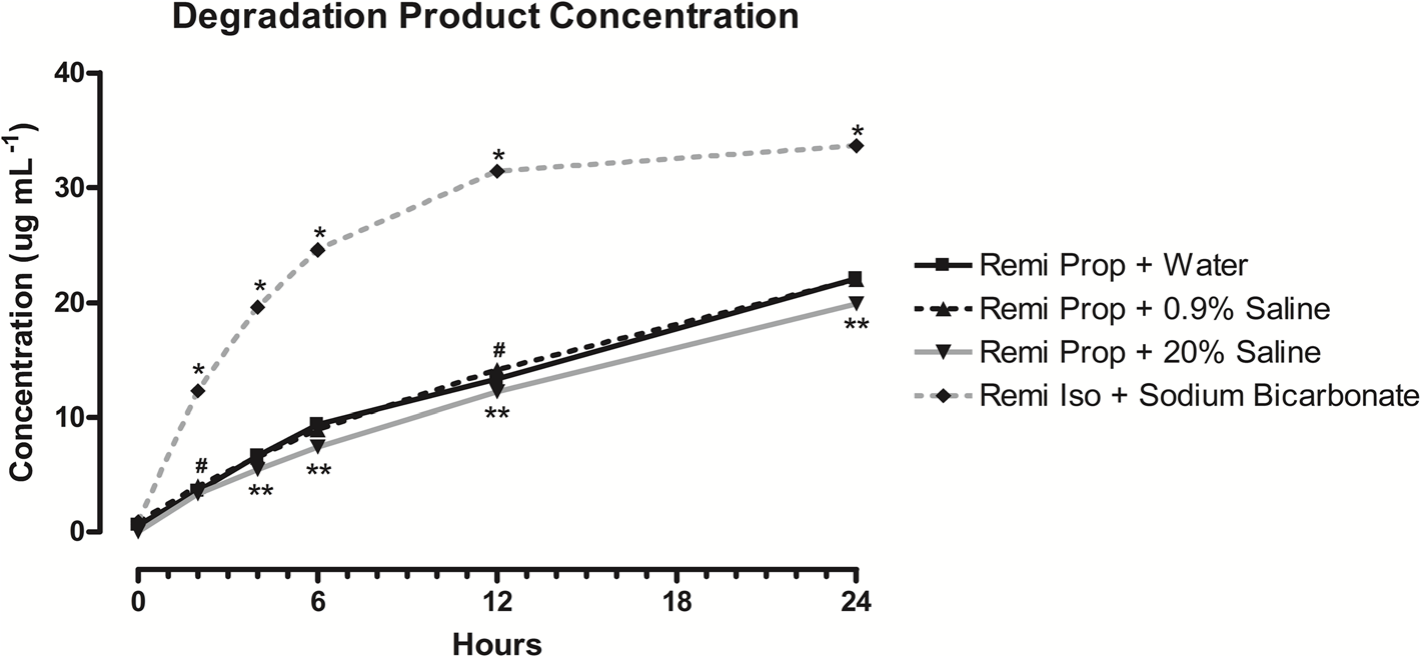
Concentration of the degradation product over 24 h in samples containing 30 μg mL^− 1^ of remifentanil reconstituted with sodium bicarbonate solution in isolation, and in samples containing 30 μg mL^− 1^ of remifentanil reconstituted with water, 0.9% saline solution, and 20% saline solution and mixed with propofol. Data expressed as mean ± SEM, n = 3. *P < 0.05 vs. water, 0.9% saline solution, and 20% saline solution; **P < 0.05 vs. water and 0.9% saline solution; ^#^*P* < 0.05 vs. water

A degradation product with an ion weight of 362 Da was formed over 24 h in all samples analysed containing remifentanil. Solutions containing 30 μg mL^− 1^ of remifentanil reconstituted with sodium bicarbonate solution in isolation produced the highest concentration of the degradation product (P < 0.0001), with 33.7 μg mL^− 1^ detectable after 24 h (Fig. 2). Interestingly, this combination also resulted in the greatest remifentanil degradation of the samples analysed. Samples containing 30 μg mL^− 1^ of remifentanil in propofol that were reconstituted with water and 0.9% saline exhibited a similar increase in the degradation product, with 22.1 μg mL^− 1^ and 22.0 μg mL^− 1^ detected after 24 h, respectively. Both solutions contained significantly more degradation product (*P* < 0.021) than 20% saline-reconstituted solutions, with 19.9 μg mL^− 1^ detected after 24 h (Fig. 2).

## Discussion

This study demonstrated that the influence of reconstitution medium, pH and drug concentration is important for the stability of a remifentanil-propofol solution. The stability of remifentanil following reconstitution is more affected by the pH of the reconstitution medium than the initial remifentanil concentration. Remifentanil degraded significantly when mixed with propofol. Concentration had more of an effect on remifentanil degradation in the mixture, with greater stability observed at higher remifentanil concentrations. The initial concentration of remifentanil was also found to impact on propofol degradation, with less stability observed as the amount of remifentanil increased. In all cases, however, the degradation seen appears to be affected by the influence of initial remifentanil concentration on solution pH, as solutions containing 50 μg mL^− 1^ of remifentanil had a lower overall pH than those containing 10 μg mL^− 1^ of remifentanil. Interestingly, these findings correspond with the suggested infusion concentration of 50 μg mL^− 1^ provided by the manufacturers of remifentanil hydrochloride. For all remifentanil solutions (isolated and mixed), elevated pH resulted in increased formation of degradation products.

A comparable study from Stewart et al. investigated the stability of high (50 μg mL^− 1^) and low (5 μg mL^− 1^) concentrations of remifentanil in 10 mg mL^− 1^ of propofol when stored in polyvinyl chloride bags and propylene syringes [22]. Similar to our results, they demonstrated that both drugs in isolation remained stable while the mixture did not, the higher remifentanil concentration had greater stability than the low concentration, propofol was more stable in isolation than when mixed with remifentanil, and the storage conditions have a greater influence on propofol stability than the initial remifentanil concentration added to the mixture [22]. Another similar study by Gersonde, Eisend, Haake and Kunze investigated the physicochemical compatibility and emulsion stability of propofol when mixed and stored with other sedatives and analgesics, including remifentanil, in a syringe for a period of 7 days [23]. All solutions were reconstituted and diluted with 0.9% NaCl, and mixed at ratios of 10:1 (v/v), 1:1 (v/v) and 1:10 (v/v) using a remifentanil concentration of 0.05 mg mL^− 1^ and a propofol concentration of 20 mg mL^− 1^. Comparable to our investigation, the study demonstrated that the concentration remained above 90% for the isolated drugs after 24 h, while the mixture containing the lowest remifentanil concentration showed the greatest change in drug concentration, decreasing to below 90% within 4 h [23]. These findings indicate that good control of the pH of the remifentanil reconstitution mixture and the use of higher concentrations of remifentanil show viability as an anaesthetic dosing regimen.

While a recent study by Bedocs, Evers and Buckenmaier III concurred with our findings that propofol alone remains stable over 24 h, in contrast, they found a mixture of propofol, ketamine and remifentanil stored in polypropylene tubes also showed no signs of degradation [5]. Ketamine is prepared in a slightly acidic solution of pH 3.5–5.5 [33], and its concentration in the mixtures was 200-times that of remifentanil; unfortunately, the pH of the mixtures was not determined in the study by Bedocs, Evers and Buckenmaier III, and a reduction in solution pH may have contributed to the stability seen with remifentanil in the mixtures. Our study differs from those mentioned in that we stored the solutions in glass and investigated a greater variety of reconstitution solutions and remifentanil concentrations, examining the effect on both remifentanil and propofol stability when stored in isolation and when mixed.

The effect of altering the pH of reconstituted remifentanil was investigated via the use of different reconstitution mediums that were chosen due to their pH characteristics, rather than their physiological properties or use in pharmaceutical reconstitution. However, manufacturers of remifentanil recommend both sterile water for injection and 0.9% sodium chloride injection for reconstitution.

Remifentanil reconstituted with 8.4% sodium bicarbonate solution in isolation had an average pH of 8.7 over 24 h for all concentrations examined, and resulted in the concentration of remifentanil decreasing rapidly. This was expected, due to the rapid aqueous hydrolysis of the sterically unhindered alkyl ester that occurs at high pH [34]. Conversely, remifentanil in isolation that was reconstituted with water, 0.9% saline solution, and 20% saline solution all had an average pH below 4 over 24 h for all concentrations tested. The remifentanil in these solutions was very stable, with over 90% of the original concentration remaining after 24 h. These results are consistent with known remifentanil pharmaceutics and confirm the role of pH in its degradation. Furthermore, it highlights the importance of considering the pH of any pharmaceutical that is to be mixed with remifentanil. Due to the instability of the remifentanil-sodium bicarbonate solution, it was not included in the remifentanil-propofol mixture stability study.

While remifentanil degraded significantly when mixed with propofol, those solutions reconstituted with 20% saline were found to be the most stable over 24 h for all concentrations tested, significantly so for 40 and 50 μg mL^− 1^ concentrations. We believe this is due to the pH of these solutions, as they had the lowest of all reconstitution solutions tested and were the only solutions to have an average pH below 7. Furthermore, remifentanil mixed with propofol was most stable when the solution pH was below pH 6, particularly around pH 5.7. This demonstrates that pH is an important factor in remifentanil stability not only in isolation, but also when it is mixed with propofol; however, it cannot be concluded that pH is the only factor influencing remifentanil stability in the mixture.

**Fig. 3:**
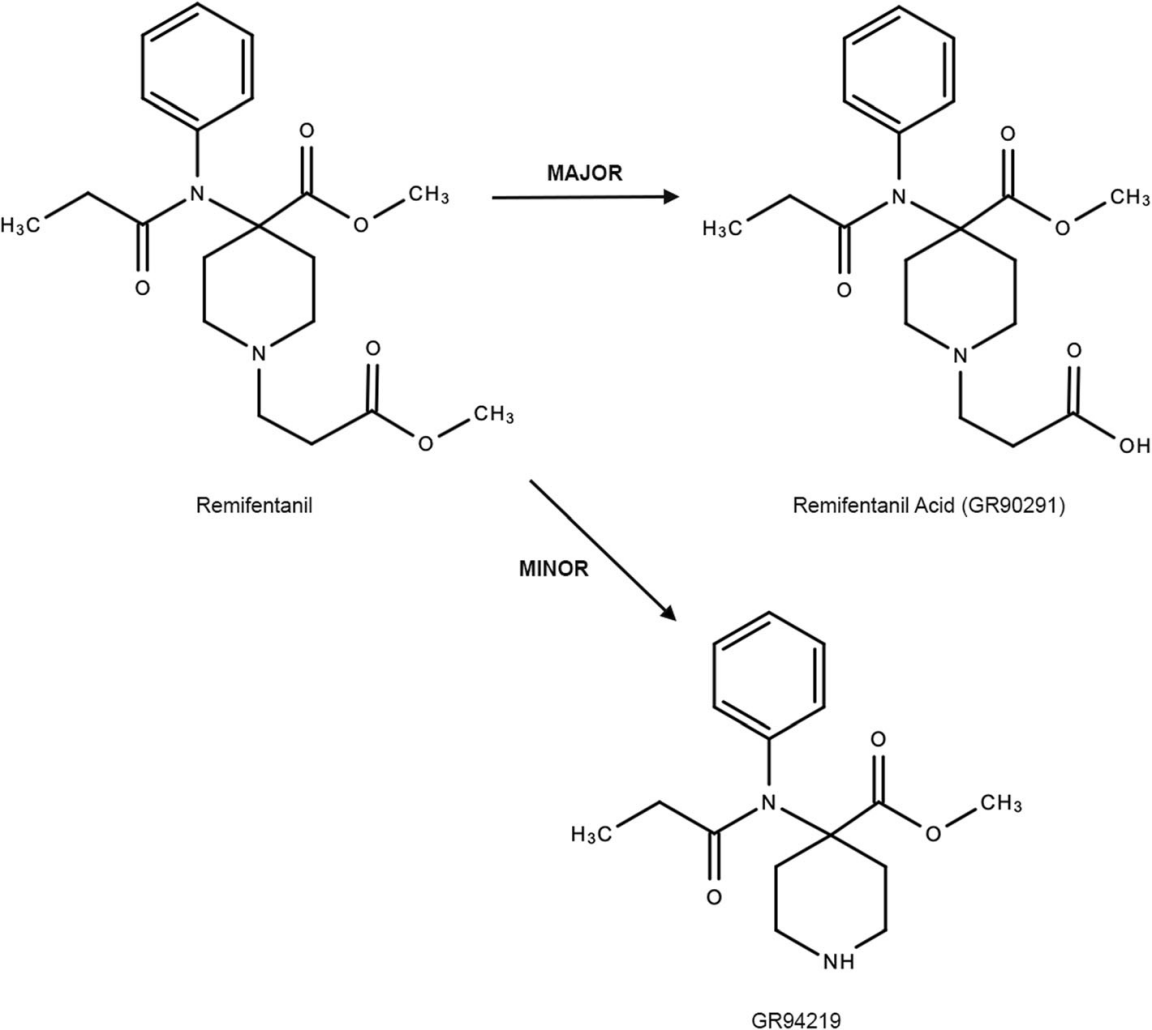
Metabolic pathway of remifentanil showing its major metabolite, remifentanil acid (GR90291), and a minor metabolite (GR94219). Modified from Westmoreland et al. [35]

We examined the solutions that demonstrated the highest degradation of remifentanil. A mid-range remifentanil concentration of 30 μg mL^− 1^ was chosen for further analysis as those samples showed sufficient degradation in the previous studies. Remifentanil was visible via mass spectrometry when run in the positive ion mode, with an ion weight of 376 Da, while the degradation product had an ion weight of 362 Da. It was found that the increase in concentration of the degradation product over 24 h was directly proportional to both the alkalinity of the solution and the degradation of remifentanil. These findings correspond to those reported by Gersonde, Eisend, Haake and Kunze [23] and suggest that the detected by-product is a degraded form of remifentanil. Due to the ion weight of the unknown compound and the rapid de-esterification experienced by remifentanil, it is speculated that this degradation product is the principal metabolite, remifentanil acid (GR90291; Fig. 3) [9, 36, 37]. Although this metabolite, eliminated by the kidneys, may accumulate in patients with severe renal impairment [9], it is much less potent (1/4600) than its parent compound [37] and does not result in clinically-significant prolonged mu-opioid effects [38]. A minor metabolite of remifentanil, the β-elimination product (GR94219), is also produced at high pH through the “retro-Michael reaction”; however, the dominant and rapid esterase metabolism results in only approximately 1% of remifentanil being eliminated in this secondary form (Fig. 3) [39]. While these degradation products may not be pharmacologically relevant for most patients, their formation does render the mixture less effective in a clinical setting and highlights the importance of understanding the chemistry associated with mixing compounds.

When in isolation and when mixed with water, 0.9% saline solution or 20% saline solution, propofol concentration remained above 90% over 24 h regardless of diluent concentration. This is potentially important in the clinical setting, as it indicates that propofol concentration remains stable over prolonged periods of infusion.

While propofol concentration remained above 90% in all solutions tested, those containing the highest concentration of saline (20%) at the highest volume (5 mL) resulted in the greatest propofol degradation, even in isolation. These findings are supported by a previous study by Wei et al., who demonstrated that propofol was stable for six hours following dilution with large volumes of sodium chloride (ranging from 500 mL to 800 mL in a total of 1000 mL, much greater volumes than those used in our study) [40].

Nemec, Germ, Schulz-Siegmund and Ortner found that the addition of 0.9% sodium chloride to propofol 1% in the ratio of 1:1 (v/v) resulted in a minor change in the emulsion stability [41]. Emulsions containing phospholipids such as egg lecithin are charge-stabilised and have a zeta potential that enables excellent stability under normal conditions. One factor that may lower the zeta potential and impact emulsion stability is the presence of electrolytes [30]. It has been suggested that the addition of positively-charged sodium results in neutralisation of the negatively-charged surface of the propofol emulsion oil droplets, resulting in flocculation [40]. This may explain the decreased propofol stability, albeit minor, that was observed following the addition of 20% saline in our study. It should be noted that a combination of propofol and saline solution has been investigated for use in several clinical applications, including reducing pain on injection and decreasing the incidence and severity of excitatory reactions during induction of anaesthesia in young children [42, 43].

While the large decrease in solution pH following the addition of remifentanil did not have a significant impact on propofol concentration specifically, pH may also affect the zeta potential, and therefore stability, of phospholipid-stabilised emulsions [44]. Therefore, the decrease in solution pH experienced following the addition of both 20% saline solution (in isolation) and remifentanil may have contributed to minor propofol emulsion destabilisation, even in mixtures where remifentanil was reconstituted with water; this may be confirmed by reconstituting remifentanil directly into the propofol emulsion. Furthermore, the decrease in pH observed over 24 h in remifentanil-propofol mixtures may be partly attributed to the release of small amounts of free fatty acids from the propofol emulsion, as a result of phospholipid and soybean oil hydrolysis [32].

Due to the concentration degradation experienced in the remifentanil-propofol mixture, further analyses of stability, such as emulsion fat globule size/distribution, were not deemed necessary. Furthermore, these factors have been investigated in previous studies [23, 41, 45].

## Conclusions

It is clear the stability of remifentanil is less dependent on the initial concentration and more influenced by the pH of the solution, as the addition of a neutral/alkali diluent had a negative impact on its stability. Additionally, mixing remifentanil with propofol in the same storage vessel resulted in significant remifentanil degradation. The hydrolysis of remifentanil at more alkaline pH values is likely a factor in the degradation observed in our study, and the results suggest that reconstituting remifentanil in a solution with a more acidic pH may increase its short-term storage stability. For propofol, the addition of remifentanil, water or saline solution at the concentrations tested, as well as the resulting changes in pH, did not have a significant negative impact on its concentration when stored over 24 h. However, our study indicates, from a chemistry perspective, that remifentanil and propofol may not be suitable to store as an admixture long-term prior to infusion.

## Supplementary Information

**Additional file 1.** Additional Table 1 A table containing the pH of remifentanil solutions reconstituted with water, 0.9% or 20% saline solution, or sodium bicarbonate solution over 24 h.

## Data Availability

The datasets used and/or analysed during the current study are available from the corresponding author on reasonable request.

## Abbreviations

HPLC: High Performance Liquid Chromatography
IV: Intravenous
MS: Mass Spectrometry
GABA: Gamma-Aminobutyric Acid
ANOVA: Analysis of Variance
