# Supplementary material for: The effect of concentration, reconstitution solution and pH on the stability of a remifentanil hydrochloride and propofol admixture for simultaneous co-infusion
Source: BMC Anesthesiol. 2020 Nov 12;20:283. doi: 10.1186/s12871-020-01194-5 (PMC7664089; doi:10.1186/s12871-020-01194-5)
Supplement: Supplementary file 1 — Additional file 1. Additional Table 1 A table containing the pH of remifentanil solutions reconstituted with water, 0.9% or 20% saline solution, or sodium bicarbonate solution over 24 h. [file 12871_2020_1194_MOESM1_ESM.doc]

**Additional Table 1.** pH of remifentanil over 24 hours when reconstituted with water, saline, or sodium bicarbonate solution

|  | **10 µg mL^-1^** | | | |  | **20 µg mL^-1^** | | | |  | **30 µg mL^-1^** | | | |  | **40 µg mL^-1^** | | | |  | **50 µg mL^-1^** | | | |
| --- | --- | --- | --- | --- | --- | --- | --- | --- | --- | --- | --- | --- | --- | --- | --- | --- | --- | --- | --- | --- | --- | --- | --- | --- |
|  | **1 h** | **6 h** | **12 h** | **24 h** |  | **1 h** | **6 h** | **12 h** | **24 h** |  | **1 h** | **6 h** | **12 h** | **24 h** |  | **1 h** | **6 h** | **12 h** | **24 h** |  | **1 h** | **6 h** | **12 h** | **24 h** |
| **pH of Solution (mean ± SEM; n=3)** | | | | | | | | | | | | | | | | | | | | | | | | |
| **W** | 3.67  ±0.04  ^**^ | 3.69  ±0.02  ^**, e^ | 3.73  ±0.01  ^**^ | 3.68  ±0.01  ^**^ |  | 4.01  ±0.03  ^e^ | 3.90  ±0.01  ^**, e^ | 3.92  ±0.04  ^, e | 3.85  ±0.03  ^**, a^ |  | 3.86  ±0.02  ^a, d^ | 3.75  ±0.01  ^**^ | 3.72  ±0.03  ^ | 3.77  ±0.01  ^**, a^ |  | 3.71  ±0.09  ^b^ | 3.80  ±0.00  ^**, b, d^ | 3.82  ±0.00  ^e^ | 3.82  ±0.04  ^a^ |  | 3.48  ±0.06  ^**^ | 3.63  ±0.01  ^b^ | 3.58  ±0.01  ^**, e^ | 3.67  ±0.08 |
| **0.9%** | 4.40  ±0.03  ^e^ | 4.29  ±0.01  ^e^ | 4.49  ±0.02  ^e^ | 4.42  ±0.04  ^e^ |  | 4.03  ±0.01  ^e^ | 3.99  ±0.01  ^, e | 3.82  ±0.03  ^ | 4.16  ±0.02  ^e^ |  | 3.92  ±0.01  ^ | 3.87  ±0.01 | 3.76  ±0.01  ^ | 3.97  ±0.04 |  | 3.77  ±0.03  ^b^ | 3.72  ±0.01  ^b, d^ | 3.87  ±0.01  #, ^, b | 3.82  ±0.01  ^, b, d |  | 3.71  ±0.00  ^, b | 3.66  ±0.01  ^, b | 3.69  ±0.01  ^, e | 3.67  ±0.01  ^, b |
| **20%** | 4.37  ±0.01  ^e^ | 4.33  ±0.02  ^e^ | 4.39  ±0.03  ^e^ | 4.40  ±0.04  ^e^ |  | 4.05  ±0.01  ^e^ | 4.04  ±0.02  ^e^ | 4.09  ±0.02  ^e^ | 4.16  ±0.03  ^e^ |  | 3.85  ±0.00 | 3.85  ±0.01 | 3.89  ±0.01 | 3.92  ±0.01 |  | 3.77  ±0.01  ^b, d^ | 3.75  ±0.00  ^b, d^ | 3.81  ±0.01  ^b, d^ | 3.76  ±0.01  ^b, d^ |  | 3.67  ±0.00  ^b^ | 3.60  ±0.01  ^b^ | 3.74  ±0.01  ^b^ | 3.71  ±0.00  ^b^ |
| **SB** | 8.64  ±0.02  ^*, d^ | 8.62  ±0.03  ^*^ | 8.79  ±0.02  ^*, d^ | 8.82  ±0.02  ^*, d^ |  | 8.56  ±0.01  ^*, a, c^ | 8.63  ±0.00  ^*, b, d^ | 8.73  ±0.03  ^*, c^ | 8.82  ±0.03  ^*^ |  | 8.57  ±0.00  ^*, a, c^ | 8.64  ±0.00  ^*, d^ | 8.75  ±0.02  ^*, c, d^ | 8.85  ±0.02  ^*, d^ |  | 8.62  ±0.01  ^*^ | 8.68  ±0.02  ^*, d^ | 8.84  ±0.01  ^*^ | 8.85  ±0.01  ^*, d^ |  | 8.55  ±0.01  ^*, c^ | 8.58  ±0.01  ^*^ | 8.67  ±0.01  ^*, c^ | 8.75  ±0.01  ^*^ |

W = water, 0.9% = 0.9% saline, 20% = 20% saline, SB = sodium bicarbonate

^*^P<0.01 vs. water, 0.9% saline solution and 20% saline solution; ^#^P<0.04 vs. water; ^P<0.05 vs. 20% saline solution; ^**^P<0.05 vs. 0.9% saline solution and 20% saline solution

^a^P<0.05 vs. 10 µg mL^-1^; ^b^P<0.05 vs. 30 µg mL^-1^; ^c^P<0.04 vs. 40 µg mL^-1^; ^d^P<0.05 vs. 50 µg mL^-1^; ^e^P<0.05 vs. all other concentrations
